# Band-selective NMR experiments for suppression of unwanted signals in complex mixtures†

**DOI:** 10.1039/d0ra06828d

**Published:** 2020-09-01

**Authors:** Elin Alexandersson, Corine Sandström, Lena C. E. Lundqvist, Gustav Nestor

**Affiliations:** Department of Molecular Sciences, Swedish University of Agricultural Sciences Uppsala Sweden gustav.nestor@slu.se

## Abstract

Band-selective NMR experiments are presented that allow selective suppression of unwanted signals (SUN) from the spectra of complex metabolite mixtures. As a result, spectral overlap and dynamic range problems are substantially reduced and low-intensity signals normally covered by dominant signals can be observed. The usefulness of the experiments is exemplified with selective suppression of sugar signals from the NMR spectra of fruit juice and a plant sample. Other possible applications include blood, milk, and wine samples.

## Abstract 2

Band-selective NMR experiments are presented that allow selective suppression of unwanted signals (SUN) from the spectra of complex metabolite mixtures.

## Graphical abstract

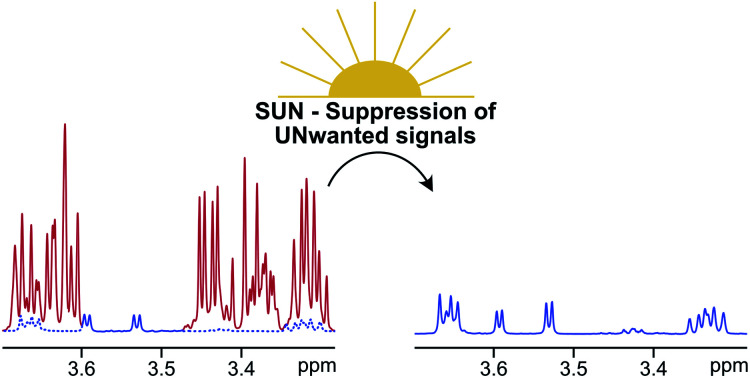

## Introduction

Biological samples typically contain numerous different metabolites with large variations in concentration, where certain compounds are present in considerably higher concentration than others. This complexity causes severe spectral overlap and dynamic range problems when the samples are analysed by NMR spectroscopy. For instance, NMR spectra of plant extracts, fruit juices, blood, milk, and tissues are typically dominated by sugars such as glucose, fructose, sucrose, and lactose, meaning that other metabolite signals in the spectral region ∼3–5 ppm are obscured by the sugar signals. Sample pre-treatment, *e.g.* chromatography, is therefore often needed before the NMR analysis to enable low-abundant metabolites to be studied. Alternative ways to study complex mixtures by NMR have been developed. One method is to record a second spectrum of the sample where an extra amount of the abundant metabolite has been added and then calculate the difference between the two spectra.^1^ However, this requires sample manipulation, highly stable conditions, and extensive spectral fitting. Another way to remove carbohydrates is to chemically degrade them by adding an oxidative agent to the sample before analysis.^2^ Although allegedly efficient, this strategy is irreversible and might break down certain non-carbohydrate molecules as well. Other approaches include methods based on band-selective excitation of the spectral area of interest^3,4^ (often combined with statistical analyses^5^), computational methods,^6^ and selective experiments utilizing differences in relaxation, diffusion, and *J*-coupling, combined with mathematical modelling.^7,8^

Most of the cited strategies require statistical and computational analyses and/or that the sample is altered in some way. Our aim was to develop an NMR-based approach requiring minimal sample manipulation and computational work that still gives information about as many compounds in a mixture as possible. In 1999, Rutherford *et al.* suggested an NMR experiment for selective removal of benzylic methylene signals from the spectra of benzyl ether-protected carbohydrates.^9^ The method is based on the excitation sculpting pulse sequence^10^ and uses band-selective pulses to defocus all signals in a selected region of the spectrum. This first step is followed by a TOCSY spin-lock that restores any signal in the selected region that is *J*-coupled to a signal not affected by the excitation sculpting sequence. 2D extensions of this method were later developed for the same purpose^11^ and the principle, albeit with a different pulse sequence, has also been employed to selectively suppress signals from water^12^ and polyethylene glycol.^13^

We show here that modified versions of the original pulse sequences^9,11^ can efficiently be used to selectively remove signals from dominant compounds, *e.g.* sugars, in the spectra of complex metabolite mixtures. Thereby, signals that are otherwise hidden by the dominant signals can be observed. We call the approach SUN, Suppression of UNwanted signals. The experiments can be applied to any type of sample where certain compounds are present in excess and cause spectral overlap and/or dynamic range problems.

## Results and discussion

**Fig. 1:**
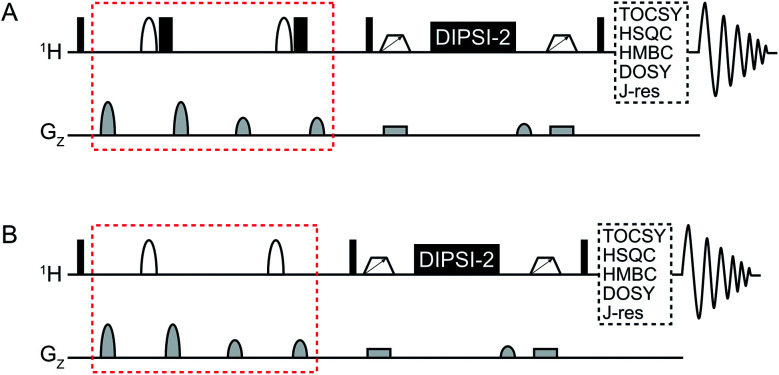
General overview of the SUN pulse sequences used for (A) band-selective suppression and (B) band-selective excitation. The difference between the two versions is marked in red. Narrow black rectangles denote 90° hard pulses while wide black rectangles denote 180° hard pulses. Shaped white bars represent band-selective inversion pulses. White trapezoids with arrows denote chirp pulses aimed for suppression of zero-quantum coherences.^15^ DIPSI-2 is used for isotropic mixing.^14^

An overview of the pulse sequences used in this work is shown in Fig. 1. The first one (Fig. 1A) is a modified version of the methods described above.^9,11^ The second pulse sequence (Fig. 1B) utilizes the opposite strategy, *i.e.* band-selective excitation of the regions of the spectrum that do not contain the metabolite(s) to be suppressed. The double-pulsed field gradient spin-echo (DPFGSE) suppression or excitation is followed by a TOCSY spin-lock, here DIPSI-2 ^14^ with zero-quantum coherence suppression.^15^ As indicated in the figure, the pulse sequences can be used in conjunction with *e.g.* TOCSY, HSQC, or HMBC to obtain various 2D experiments with band-selective suppression or excitation. If desired, the band-selective inversion pulse can be designed to target several regions of the spectrum at the same time. It is crucial that all signals from the dominant compound(s) are suppressed in the DPFGSE step; otherwise they will also be restored during the spin-lock.

**Fig. 2:**
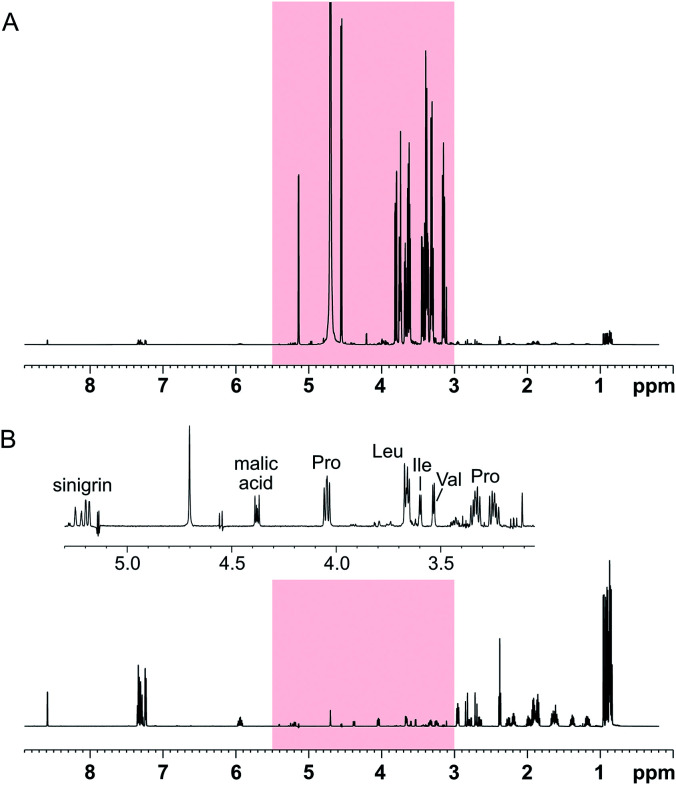
SUN applied to an artificial mixture containing 100 mM d-glucose and 1 mM of proline, leucine, isoleucine, valine, histidine, phenylalanine, γ-aminobutyric acid, choline, malic acid, citric acid, ascorbic acid, and sinigrin. (A) 1D-^1^H spectrum where the region targeted for band-selective suppression is highlighted in red, (B) 1D-SUN with suppression of the region 3.0–5.5 ppm. The inset shows an expansion of the region targeted by the band-selective pulse.

The performance of the SUN approach was evaluated using an artificial mixture containing twelve common plant metabolites (proline, leucine, isoleucine, valine, histidine, phenylalanine, γ-aminobutyric acid, choline, malic acid, citric acid, ascorbic acid, and sinigrin) in equal concentrations and glucose in higher concentration. The ratio between glucose and the other metabolites was varied between 10 : 1 and 1000 : 1. Using SUN with band-selective suppression, it was possible to suppress the glucose signals almost entirely (≥98%) (Fig. 2). Several other signals in the area were retained, including the alpha protons of proline, valine, leucine, and isoleucine, of which the latter two were previously buried under the glucose signals (Fig. 2B).

**Fig. 3:**
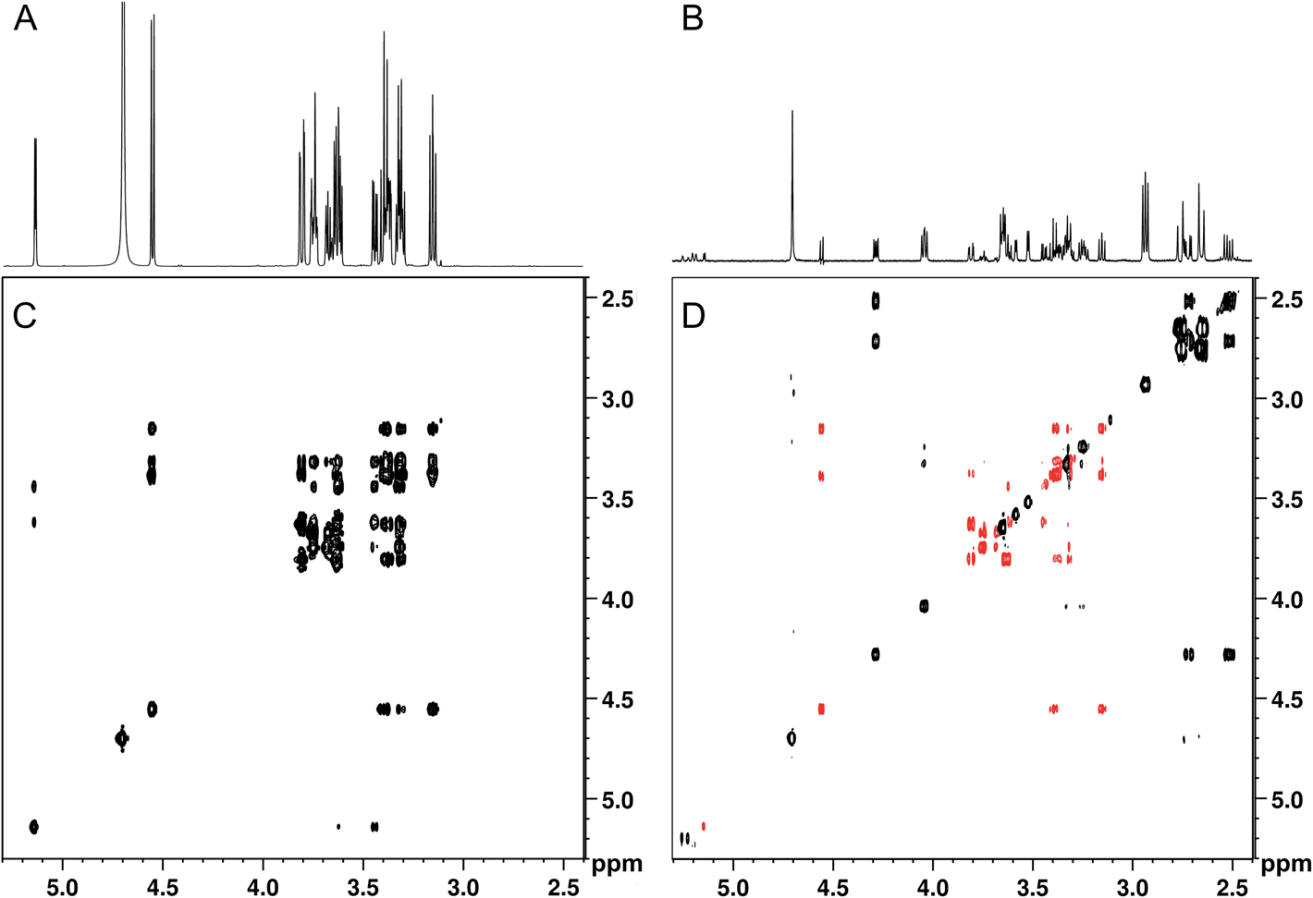
SUN applied to an artificial mixture containing 100 mM d-glucose and 0.1 mM of proline, leucine, isoleucine, valine, histidine, phenylalanine, γ-aminobutyric acid, choline, malic acid, citric acid, ascorbic acid, and sinigrin. (A) 1D-^1^H spectrum, (B) 1D-SUN with excitation of the spectral regions 5.8–8.6 ppm and −0.2-2.6 ppm, (C) 2D-TOCSY, (D) 2D-SUN-TOCSY with excitation of the same regions as in (B) and remaining glucose signals coloured red.

When band-selective excitation was used, almost identical results were obtained in terms of glucose suppression, resolution, and signal-to-noise ratio (Fig. 3A and B) (note that Fig. 2 shows a mixture with the proportion 100 : 1 whereas it is 1000 : 1 in Fig. 3). However, the performance of this pulse sequence appeared to be more sensitive to the exact positioning of the selective pulse than the version with band-selective suppression. Thus, both the width of the spectral regions chosen for excitation and their location relative to each other highly influenced the suppression efficiency. The best suppression of glucose signals was obtained when the two spectral regions excited simultaneously were of the same width.

Although spectral overlap is less of a problem in two-dimensional spectra, abundant compounds can still prevent identification of other compounds present in lower concentration. Therefore, 2D analogues of the SUN pulse sequences were developed, including TOCSY, HSQC, HMBC, DOSY, and *J*-resolved spectroscopy with band-selective suppression or excitation. The performance of the 2D experiments is illustrated with 2D-SUN-TOCSY applied to the 1000 : 1 artificial mixture, carried out using band-selective excitation to selectively remove glucose (Fig. 3C and D). An HSQC version was evaluated as well (see Fig. S2†). It is apparent that the glucose suppression obtained in the 2D experiments highly resembles that of their 1D counterparts. Apart from the possibility of detecting analytes in the glucose region, the improved receiver gain achieved both in the 1D and 2D experiments when suppressing the glucose signals significantly improves identification of low-concentration analytes throughout the spectra.

As expected, the length of the TOCSY spin-lock and the size of the *J*-coupling between the suppressed signals and their non-suppressed neighbours determine to what extent signals are recovered in the TOCSY step. Since signal intensity is also influenced by the relaxation rate of the individual spins, a compromise might be needed so that the mixing time is long enough for TOCSY transfer to take place, but sufficiently short to avoid signal attenuation due to relaxation. In the examples presented here, the intensity of the recovered signals did not accurately reflect the actual concentration of the compounds, meaning that quantitative analyses may require calibration curves to determine the correlation between concentration and signal intensity.

Since only signals that are *J*-coupled to another signal located outside the targeted area can be reintroduced by the TOCSY step, some non-glucose signals are missing from the band-selective spectra presented in Fig. 2 and 3. For instance, the non-aromatic protons of histidine and phenylalanine were not restored during the spin-lock. To obtain a more specific suppression of glucose with minimal impact on the other compounds, a band-selective inversion pulse targeting two separate areas of the spectrum – 3.1–3.9 ppm (the glucose ring protons) and 4.5–5.3 ppm (the anomeric protons) – was used. Thereby, additional signals were retained, both in the area in-between the glucose regions and directly underneath the glucose signals (see Fig. S3†). Non-aromatic protons belonging to histidine and phenylalanine could then be identified, as well as the methylene protons from choline. Unfortunately, suppressing two separate areas produced more severe phase distortions, mainly affecting glucose, than with only one area targeted. Both the suppression efficiency and the phase distortions were affected by the width and position of the selective pulse, as well as the pulse phase. Similar to when band-selective excitation was used, the best results were obtained when the two targeted regions were of equal size.

**Fig. 4:**
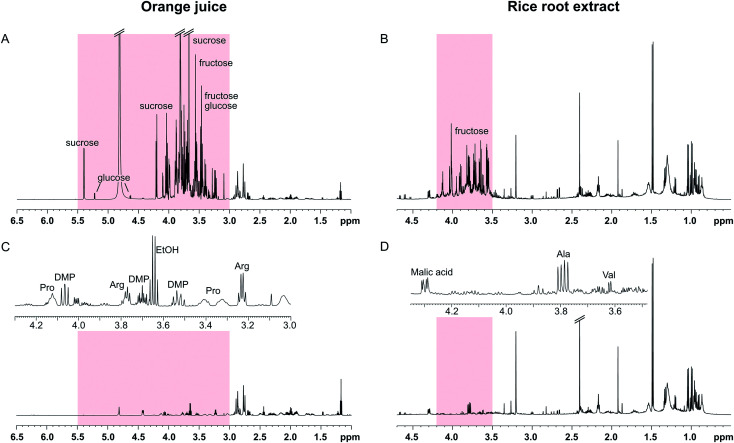
SUN applied to orange juice (left) and a rice root extract (right). (A) 1D-^1^H spectrum of orange juice recorded using excitation sculpting to suppress the water signal. (B) 1D-^1^H spectrum of a rice root extract recorded using water presaturation. (C) 1D-SUN spectrum of orange juice with band-selective suppression of the region 3.0–5.5 ppm (highlighted in red). The inset shows an expansion of the sugar ring proton region 3.0–4.3 ppm. DMP = dimethylproline. (D) 1D-SUN spectrum of the rice root extract with band-selective suppression of the region 3.5–4.2 ppm (highlighted in red). The inset shows an expansion of the fructose region 3.45–4.35 ppm.

The SUN experiments were also applied to two authentic samples: orange juice and a root extract from rice (Fig. 4). Fruit juices are complex, sugar-rich mixtures that are often analysed by NMR for quality control reasons.^16,17^ As can be seen in Fig. 4A, the orange juice spectrum was dominated by sucrose, fructose, and glucose. The sugar signals could be completely suppressed using the SUN pulse sequences which enabled identification of other metabolites in the sugar region, including ethanol, arginine, and dimethylproline (Fig. 4C). These metabolites were identified based on 1D and 2D experiments (see Fig. S4†) as well as already published orange juice signal assignments.^17–19^ It is also worth noting that the water signal was highly reduced in the band-selective experiment, even though no additional water suppression was used.

The other sample had been collected from the soil surrounding a rice plant root. Plant roots excrete a wide array of different compounds into the soil, including sugars, organic acids, and amino acids.^20^ In the spectra of the sample used here, the sugar region was dominated by fructose (Fig. 4B). Although not present in large excess, the fructose signals obstructed identification of other metabolites in the region 3.4–4.0 ppm. Since the fructose signals are all concentrated to a narrow part of the spectrum, a selective pulse targeting only this region was used in the SUN experiment. The water signal is residing outside of this region, and therefore a presaturation step was added to the pulse sequence for water suppression. The resulting spectrum is shown in Fig. 4D. With fructose removed, the signals belonging to the alanine and valine alpha protons became clearly visible (Fig. 4D). Two-dimensional TOCSY was performed for signal assignment, with similar fructose suppression as in the 1D experiment.

## Conclusions

Here we have presented two NMR experiments that are promising for suppression of unwanted signals in the spectra of complex mixtures. Using the SUN approach, the intensity of dominant signals can be dramatically reduced or completely suppressed while other signals in the targeted spectral regions are retained *via J*-coupling. Thus, problems caused by spectral overlap and a too large concentration range can be solved without physically altering the sample. In the most favourable cases, the approach can be used to identify compounds whose signals outside of the suppressed area are highly overlapped. The experiments are fast and easy to use and can be readily applied to practically any sample that contains high-intensity signals localized to just one or a few regions of the NMR spectrum. Samples where this approach would be advantageous include blood plasma, fruit juices, and different plant extracts. Both SUN versions work well to suppress sugar signals and the choice of which one to use depends on the experimental aim and sample type. We believe that both versions can become valuable tools in the study of complex mixtures.

## Experimental

### Sample preparation

#### Artificial mixtures

The mixtures were prepared with the following compounds (all purchased from Merck): d-glucose, dl-proline, l-leucine, l-isoleucine, l-valine, l-phenylalanine, l-histidine, γ-aminobutyric acid, choline chloride, malic acid, citric acid, ascorbic acid, and sinigrin hydrate. The concentration of d-glucose was either 100 mM or 1000 mM while the concentration of the other compounds was 10 mM, 1 mM, or 0.1 mM. All samples were prepared in D_2_O with a final volume of 600 μl.

#### Orange juice

Orange juice (1 ml) bought at a local supermarket was centrifuged at 13 500 rpm for 5 minutes. 540 μl of the supernatant was then mixed with 60 μl D_2_O in an NMR tube.

#### Rice root sample

The root from an eight-week old rice plant was removed from the soil and vortexed for 1 min in 30 ml MilliQ water, after which the solution was freeze-dried. 100 mg of the freeze-dried material was ultra-sonicated together with 8 ml methanol for 10 min and then centrifuged for 10 min at 5000 rpm. The supernatant was freeze-dried, after which 380 μl MilliQ water was added. After vortexing, the sample was ultra-sonicated for 10 min and then centrifuged for 10 min at 5000 rpm. 350 μl of the supernatant was then mixed with 50 μl D_2_O, 20 μl MilliQ water, 150 μl 0.4 M phosphate buffer (pH 7), and 30 μl TSP internal standard (5.8 mM). To concentrate the sample further, two identical samples prepared as described above were pooled together, freeze-dried, and then dissolved in 150 μl D_2_O for NMR analysis.

### NMR experiments

All spectra were acquired on a Bruker Avance III 600 MHz spectrometer with a 5 mm ^1^H/^13^C/^15^N/^31^P inverse detection cryoprobe equipped with a *z* gradient with a maximum nominal gradient strength of 48.1 G cm^−1^. Spectra were recorded at 25 °C and were processed with TopSpin 4.0.6. The ^1^H spectral window was set to 9 ppm or 12 ppm and the ^13^C spectral window was set to 110–122 ppm (HSQC). The carrier frequency was placed on the water signal (4.70 ppm). Further details about the NMR experiments are provided in the ESI.†

## Conflicts of interest

There are no conflicts to declare.

## Supplementary Material

RA-010-D0RA06828D-s001
