# Supplementary material for: Band-selective NMR experiments for suppression of unwanted signals in complex mixtures
Source: RSC Adv. 2020 Sep 1;10(54):32511–5. doi: 10.1039/d0ra06828d (PMC9056649; doi:10.1039/d0ra06828d)
Supplement: RA-010-D0RA06828D-s001 [file RA-010-D0RA06828D-s001.pdf]

Electronic Supplementary Material (ESI) for RSC Advances.
This journal is © The Royal Society of Chemistry 2020

Band-selective NMR experiments for suppression of unwanted signals in complex mixtures

Elin Alexandersson, Corine Sandström, Lena C.E. Lundqvist and Gustav Nestor*

Department of Molecular Sciences, Swedish University of Agricultural Sciences, Uppsala, Sweden

Electronic supplementary information

1. Pulse sequences S2
2. Supplementary spectra
   - Figure S2 .... S5
   - Figure S3 .... S6
   - Figure S4 .... S7
3. Pulse sequences in Bruker format
   - 1D SUN with band-selective suppression/excitation S8
   - 2D SUN-TOCSY with band-selective suppression/excitation S13
   - 2D SUN-HSQC with band-selective suppression/excitation S18

1. Pulse sequences

The pulse sequences for the **1D SUN** experiments are shown in Fig. S1a and S1b. Narrow black rectangles denote 90° hard pulses while wide black rectangles denote 180° hard pulses. Shaped white bars represent band-selective inversion or excitation 180° pulses. IBURP-2 shapes were used for both suppression and excitation experiments. Typically, a 3.5 ms IBURP pulse centred in the middle of the glucose region yields a bandwidth of 1500 Hz, which is enough for suppression of glucose. The Bruker WaveMaker tool was used for construction of shaped pulses targeting more than one region of the spectrum. TOCSY transfer is achieved by using the DIPSI-2 mixing scheme with a mixing time of 10-150 ms depending on the spin system (here, 100 ms was used for the rice root sample whereas 50 ms was used for the orange juice and the artificial mixtures). White trapezoids with arrows denote low-power 180° chirp pulses of 20 kHz bandwidth aimed for suppression of zero-quantum coherences. Their durations were set to 20 and 15 ms before and after the DIPSI-2 mixing, respectively. All gradient pulses except for G_0 have a duration of 1 ms. G_1 and G_2 (amplitude of 14.9 and 5.3 G cm^{-1}, respectively) are used to eliminate magnetization of signals within the bandwidth of the selective pulse (band-selective suppression, Fig S1a) or outside of the bandwidth of the selective pulse (band-selective excitation, Fig. S1b). G_3 is a spoil gradient pulse with an amplitude of 3.4 G cm^{-1}. G_0 (amplitude of ca. 2.4 and 3.2 G cm^{-1} before and after the DIPSI-2 mixing, respectively) is a weak pulsed field gradient applied simultaneously with the chirp pulses to suppress zero quantum coherence. All gradient pulses are followed by a recovery delay of 200 μs. The phase cycle is given in Table S1.

To achieve optimal suppression of unwanted signals, it might be necessary to adjust the 90° pulse length and the precise phases of the band-selective pulses (by phase corrections). The TOCSY mixing time might also need some optimization in order to obtain the best recovery of remaining signals.

The 2D TOCSY and HSQC versions of the SUN experiments are shown in Fig. S1c and S1d. The **2D SUN-TOCSY** experiment has two TOCSY transfers, where the first is applied to restore signals in the sugar region of the spectrum and the second is applied to yield TOCSY correlations in the f1 dimension. The two TOCSY steps could be used with different mixing times, but for the examples herein, the same mixing time was used. The 2D TOCSY was run in the States-TPPI manner, with phases presented in Table S1.

The HSQC step of the **2D SUN-HSQC** experiment is almost identical to the Bruker pulse program *hsqcedetgpsisp.2*, which is a multiplicity-edited HSQC with PEP sensitivity enhancement and adiabatic inversion and refocusing pulses. Chirp pulses of 20 kHz bandwidth and 500 us duration were utilized for 180° ^{13}C inversion and composite chirp pulses of 2 ms duration for 180° ^{13}C refocusing. Delays were set to $\tau = 1.7$ ms, $\Delta = 3.45$ ms, $\Delta' = 862$ μs, and $\delta = 1.2$ ms. G_4 and G_5 amplitudes were set to 38.5 and 9.7 G cm^{-1}, respectively, with a duration of 1 ms, followed by a recovery delay of 200 μs. ^{13}C decoupling was obtained with the GARP-4 decoupling scheme. The experiment was run in the echo-antiecho mode, with gradient selection obtained by the reversal of the G_4 gradient pulse. The phase cycle is given in Table S1.

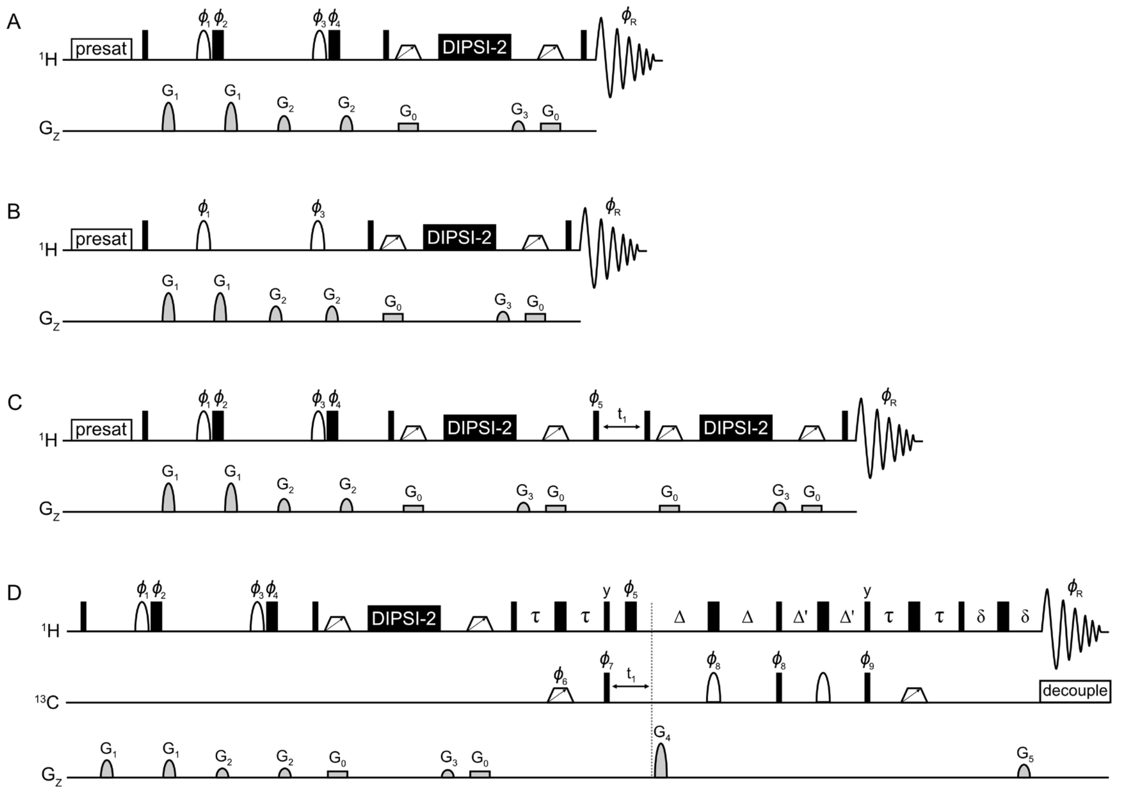

Fig. S1. Pulse sequences of (a) 1D SUN with band-selective suppression, (b) 1D SUN with band-selective excitation, (c) 2D SUN-TOCSY with band-selective suppression, (d) 2D SUN-HSQC with band-selective suppression. Phases are listed in Table S1. Unless indicated otherwise, pulses are applied along the x-axis. Band-selective excitation versions of the SUN-TOCSY and SUN-HSQC versions are obtained by eliminating the ϕ_2 and ϕ_4 180° ^1H pulses.

Table S1. Phase cycling of pulse sequences in Fig S1 (A-D).

	**A**	**B**	**C**[a]	**D**[b]
ϕ_1	x, y, -x, -y	x, y, -x, -y	x, y, -x, -y	x, y, -x, -y
ϕ_2	-x, -y, x, y	-	(-x, -y, x, y)[c]	(-x, -y, x, y)[c]
ϕ_3	x_4, y_4, $(-x)_4$, $(-y)_4$	x_4, y_4, $(-x)_4$, $(-y)_4$	x_4, y_4, $(-x)_4$, $(-y)_4$	x_4, y_4, $(-x)_4$, $(-y)_4$
ϕ_4	$(-x)_4$, $(-y)_4$, x_4, y_4	-	($(-x)_4$, $(-y)_4$, x_4, y_4)[c]	($(-x)_4$, $(-y)_4$, x_4, y_4)[c]
ϕ_5	-	-	x, -x	x_2, $(-x)_2$
ϕ_6	-	-	-	x
ϕ_7	-	-	-	x_8, $(-x)_8$
ϕ_8	-	-	-	x_2, $(-x)_2$
ϕ_9	-	-	-	y_2, $(-y)_2$
ϕ_R	$(x, -x)_2$, $(-x, x)_2$	$(x, -x)_2$, $(-x, x)_2$	x_4, $(-x)_4$	(-x), x_2, (-x), (x, $(-x)_2$, $x)_2$, (-x), x_2, (-x)

[a] Quadrature in the t_1 dimension is obtained by incrementing ϕ_5 with 90°. [b] Quadrature in the t_1 dimension is obtained by incrementing ϕ_6, ϕ_7, ϕ_9, and ϕ_R with 180°. [c] For band-selective suppression.

2. Supplementary spectra

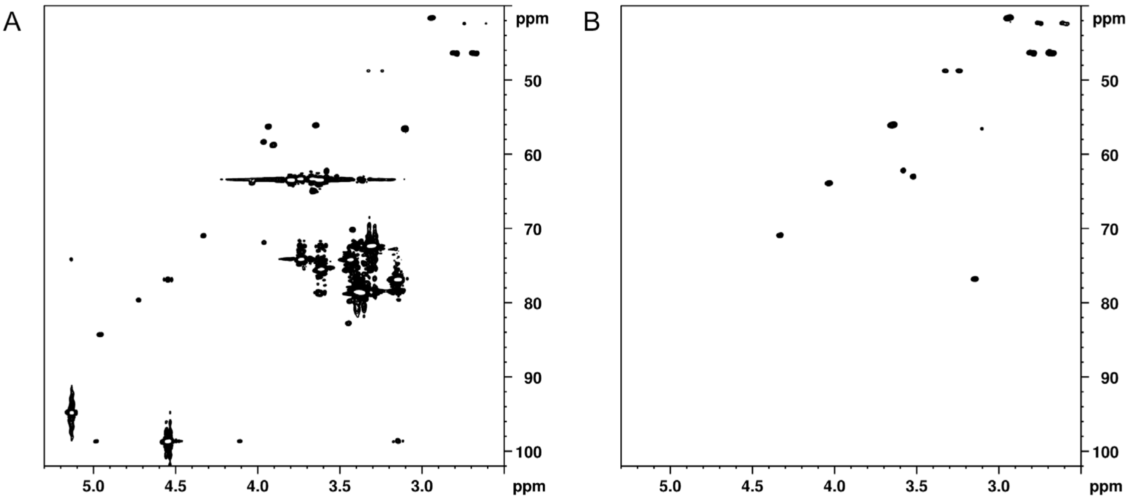

Fig. S2. (a) HSQC of the artificial mixture containing 100 mM glucose and 1 mM of the other metabolites, (b) SUN-HSQC with band-selective excitation of the spectral regions 5.8-8.6 ppm and -0.2-2.6 ppm. Data were recorded with 64 scans, 1024 data points in t_2 and 256 increments in t_1, with an experimental time of 7 hours in a) and 6.5 hours in b). The data were zero-filled before applying a $\pi/2$ shifted sine-squared bell function in both dimensions.

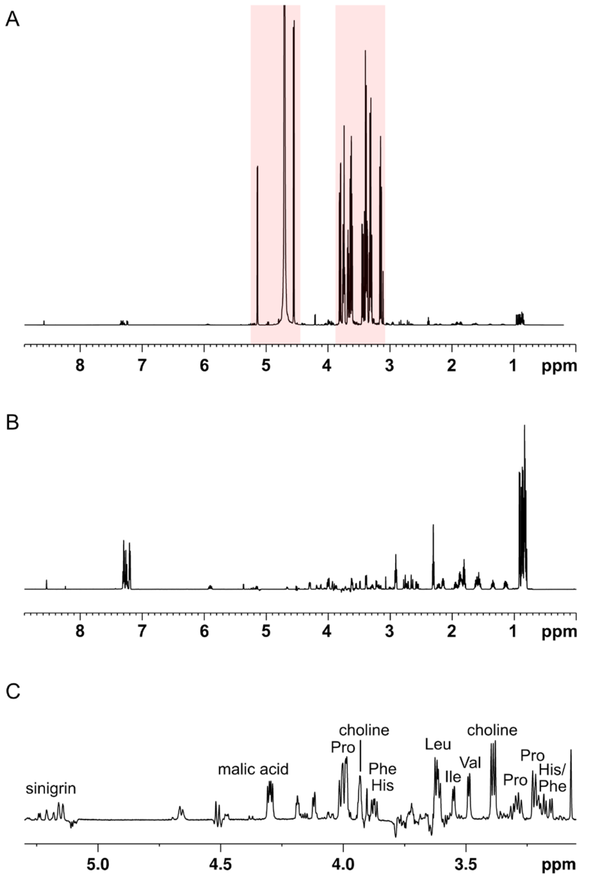

Fig. S3. 1D SUN spectra with band-selective suppression targeting two spectral regions, 4.45-5.25 ppm and 3.08-3.88 ppm. (a) 1D-^{1}H spectrum with the regions targeted for band-selective suppression highlighted in red, (b) 1D-SUN spectrum with band-selective suppression, (c) enlargement of the region 3.0-5.3 ppm in b). Data were recorded with 128 scans and 64k points, with an experimental time of 13 minutes. The data were zero-filled before applying an exponential function with 0.3 Hz line broadening.

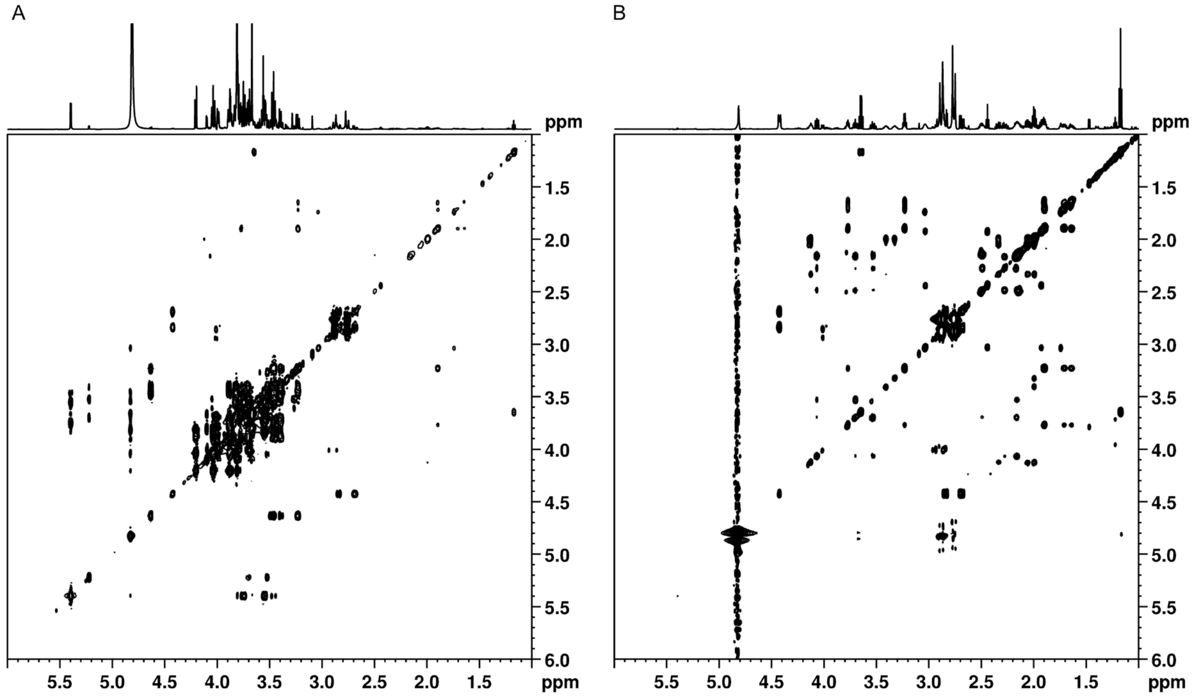

Fig. S4. 2D-TOCSY of orange juice. (a) 2D-TOCSY, (b) 2D-SUN-TOCSY with band-selective suppression of the spectral region 3.0-5.5 ppm. Data were recorded with 32 scans, 2048 data points in t_2 and 256 increments in t_1, with an experimental time of 3.5 hours. The data were zero-filled before applying a $\pi/2$ shifted sine-squared bell function in both dimensions.

3. Pulse Sequence (Bruker format)

Quick guide for starting-up

1. Copy the pulse program from this document to a text file, save it as "SUN1d" and put it in the folder named Bruker\Topspin(X.X)\exp\stan\nmr\lists\pp\user.
2. Run a ^{1}H NMR experiment and decide whether band-selective suppression or excitation will be used.
3. Define peak(s) in the middle of the desired region(s) for band-selective suppression or excitation.
4. Make a copy of the ^{1}H NMR experiment and change the pulse program to "SUN1d".
5. Set probe/solvent dependent parameters with the command "getprosol" and fill in acquisition parameters that are not automatically adjusted, such as gradient files and gradient strengths. The correct settings are provided in the pulse program.
6. Copy the peak list from the ^{1}H NMR experiment to the SUN experiment. This can be done with AU programs such as copyPL or getPL (can be found at the Bruker web library).
7. The default setting is band-selective suppression without water presaturation. To use band-selective excitation instead, add "-DEXCIT" in the ZGOPTNS field. For water presaturation, add "-DPRESAT" in the ZGOPTNS field. If both excitation and water presaturation are desired, add both ("-DEXCIT -DPRESAT") in the ZGOPTNS field.
8. Define the shape function as userA1, for instance iburp2. (For Topspin versions older than 3.6, the shape function must be defined in the pulse program. In this case, change "userA1" to the desired shape function on the line starting with "sp2:wvm".)
9. Define the bandwidth (in ppm) of the band-selective suppression or excitation region(s) as cnst18. The bandwidth is equal to the width of the selected region(s) and must be the same if more than one region is selected with the peak list.
10. Define the TOCSY mixing time (d9). Typically 50-100 ms gives efficient TOCSY transfer.
11. Run "wvm –a" to calculate the selective pulse and to add the result into the current experiment set-up.
12. Check the receiver gain with rga and start the experiment with zg.
13. If signals that should be suppressed still appear, the bandwidth for band-selective suppression (cnst18) may need to be increased to allow defocusing of the entire spin system, or the band-width for band-selective excitation (cnst18) may need to be decreased to avoid excitation of the unwanted spin system(s). In band-selective excitation, the region for excitation can also be moved away from the unwanted signals by adjusting the peak list. Adjusting p1 can also improve the suppression of unwanted signals.
14. To optimize the phase of signals in the suppressed region, if necessary, phase corrections of ph2 and ph4 may be added with the "phcor" command. Try out small changes from zero to obtain optimized phase and suppression. Also p1 can be changed to optimize the phase of the spectra. In addition, gpz0 can be adjusted to optimize the suppression of zero-quantum coherences.

For further instructions on how to use WaveMaker, we refer to the Bruker WaveMaker User Manual.

1D SUN with band-selective suppression/excitation

```
;sun1d
;
;1D suppression of unwanted signals (SUN)
;   using band-selective suppression or excitation
;   using DIPSI2 sequence for TOCSY transfer
;   with presaturation during relaxation delay (optional)
;
;Use WaveMaker for definition of shaped pulses
;Define offset(s) for shaped pulses with a peak list (PL)
;The peak list must be copied from another experiment with a macro (getPL or copyPL)
;
;Band-selective suppression without water presaturation is default
;For band-selective excitation: Use ZGOPTNS = -DEXCIT
;For presaturation during relaxation delay: Use ZGOPTNS = -DPRESAT
;
;Modified 24/06/2020
;
;           Elin Alexandersson, Corine Sandström, Lena Lundqvist and Gustav Nestor
;           Swedish University of Agricultural Sciences
;
;Avance III version
;
;$CLASS=HighRes
;$DIM=1D
;$TYPE=
;$SUBTYPE=
;$COMMENT=


#include <Avance.incl>
#include <Grad.incl>
#include <Delay.incl>


"p2=p1*2"
"d12=20u"

"spoff29=0"

"FACTOR1=(d9/(p6*115.112))/2"
"l1=FACTOR1*2"

"d2=cnst18*p1/4"

"acqt0=-p1*2/PI"


1 ze
2 30m
  d12 BLKGRAD
  d2

#   ifdef PRESAT
  d12 pl9:f1
  d1 cw:f1 ph1
  4u do:f1
#   else
  d1
#   endif /*PRESAT*/

  d12 pl1:f1
  50u UNBLKGRAD
  (p1 ph1):f1
  3u
  p16:gp1
```

```
  d16 pl0:f1
  p12:sp2:f1 ph2:r

#   ifdef EXCIT
  3u
  d12
#   else
  3u
  d12 pl1:f1
  p2 ph3
  3u
#   endif /*EXCIT*/

  p16:gp1
  d16
  3u
  p16:gp2
  d16 pl0:f1
  p12:sp2:f1 ph4:r

#   ifdef EXCIT
  3u
  d12 pl1:f1
#   else
  3u
  d12 pl1:f1
  p2 ph5
  3u
#   endif /*EXCIT*/

  p16:gp2
  d16
  p1 ph1
  3u
  3u pl0:f1
  10u gron0
  (p32:sp29 ph1):f1
  20u groff
  d16 pl10:f1

                                                            ;begin DIPSI2
3 p6*3.556 ph23
  p6*4.556 ph25
  p6*3.222 ph23
  p6*3.167 ph25
  p6*0.333 ph23
  p6*2.722 ph25
  p6*4.167 ph23
  p6*2.944 ph25
  p6*4.111 ph23

  p6*3.556 ph25
  p6*4.556 ph23
  p6*3.222 ph25
  p6*3.167 ph23
  p6*0.333 ph25
  p6*2.722 ph23
  p6*4.167 ph25
  p6*2.944 ph23
  p6*4.111 ph25

  p6*3.556 ph25
  p6*4.556 ph23
  p6*3.222 ph25
  p6*3.167 ph23
  p6*0.333 ph25
  p6*2.722 ph23
```

```
  p6*4.167 ph25
  p6*2.944 ph23
  p6*4.111 ph25

  p6*3.556 ph23
  p6*4.556 ph25
  p6*3.222 ph23
  p6*3.167 ph25
  p6*0.333 ph23
  p6*2.722 ph25
  p6*4.167 ph23
  p6*2.944 ph25
  p6*4.111 ph23
  lo to 3 times l1
                                                        ;end DIPSI2

  p16:gp3
  d16 pl0:f1
  10u gron0*1.333
  (p32*0.75:sp29 ph1):f1
  20u groff

  d16 pl1:f1
  p1 ph1

  go=2 ph31
  30m mc #0 to 2 F0(zd)
  20u BLKGRAD
exit


ph1=0
ph2=0 1 2 3
ph3=2 3 0 1
ph4=0 0 0 0 1 1 1 1 2 2 2 2 3 3 3 3
ph5=2 2 2 2 3 3 3 3 0 0 0 0 1 1 1 1
ph23=3
ph25=1
ph31=0 2 0 2 2 0 2 0


;pl0 : 0W
;pl1 : f1 channel - power level for pulse (default)
;pl9 : f1 channel - power level for presaturation
;pl10: f1 channel - power level for TOCSY-spinlock
;sp2:wvm: userA1(cnst18 ppm, PL; PA=1.0) np=1000
;sp29: f1 channel - shaped pulse (adiabatic)
;p1 : f1 channel -  90 degree high power pulse
;p2 : f1 channel - 180 degree high power pulse
;p6 : f1 channel -  90 degree low power pulse
;p12: f1 channel - 180 degree shaped pulse
;p16: homospoil/gradient pulse                    [1 msec]
;p32: f1 channel - 180 degree shaped pulse (adiabatic)    [20 msec]
;     smoothed chirp (sweepwidth, 20% smoothing, 10000 points)
;d1 : relaxation delay; 1-5 * T1
;d9 : TOCSY mixing time
;d12: delay for power switching                   [20 usec]
;d16: delay for homospoil/gradient recovery
;l1: loop for DIPSI cycle: ((p6*115.112) * l1) = mixing time
;NS: 8 * n
;DS: 4

;cnst18: effective bandwidth for shaped pulse (ppm)
;PL: peak list to define offset(s) for shaped pulse
;userA1: shape function (sp2)
```

```
;phcor 2 : phase difference between power levels sp2 and pl1
;phcor 4 : phase difference between power levels sp2 and pl1

;choose p12 and sp2 according to desired selectivity or use WaveMaker



;for z-only gradients:
;gpz0: ca. 5%
;gpz1: 31%
;gpz2: 11%
;gpz3: 7%

;use gradient files:
;gpnam1: SMSQ10.100
;gpnam2: SMSQ10.100
;gpnam3: SMSQ10.100

                                        ;preprocessor-flags-start
;EXCIT: for band-selective excitation start experiment with
;             option -DEXCIT (eda: ZGOPTNS)
;PRESAT: for presaturation during relaxation delay start experiment with
;             option DPRESAT (eda: ZGOPTNS)
                                        ;preprocessor-flags-end
```

2D SUN-TOCSY with band-selective suppression/excitation

```
;suntocsy
;
;2D suppression of unwanted signals (SUN-TOCSY)
;   using band-selective suppression or excitation
;   using DIPSI2 sequence for TOCSY transfer
;   with presaturation during relaxation delay (optional)
;
;Use WaveMaker for definition of shaped pulses
;Define offset(s) for shaped pulses with a peak list (PL)
;The peak list must be copied from another experiment with a macro (getPL or copyPL)
;
;Band-selective suppression without water presaturation is default
;For band-selective excitation: Use ZGOPTNS = -DEXCIT
;For presaturation during relaxation delay: Use ZGOPTNS = -DPRESAT
;
;Modified 18/08/2020
;
;            Elin Alexandersson, Corine Sandström, Lena Lundqvist and Gustav Nestor
;            Swedish University of Agricultural Sciences
;
;Avance III version
;
;$CLASS=HighRes
;$DIM=2D
;$TYPE=
;$SUBTYPE=
;$COMMENT=


#include <Avance.incl>
#include <Grad.incl>
#include <Delay.incl>


"p2=p1*2"
"spoff29=0"

"in0=inf1"

"d0=in0/2-p1*4/3.1416"
"d2=cnst18*p1/4"
"d11=30m"
"d12=20u"

"FACTOR1=(d9/(p6*115.112))/2"
"l1=FACTOR1*2"
"FACTOR2=(d10/(p6*115.112))/2"
"l2=FACTOR2*2"

"acqt0=-p1*2/3.1416"


1 ze
2 d11
  d2

#   ifdef PRESAT
  d12 pl9:f1
  d1 cw:f1 ph1
  4u do:f1
#   else
  d1
#   endif /*PRESAT*/

  d12 pl1:f1
```

```
  50u UNBLKGRAD
  (p1 ph1):f1
  3u
  p16:gp1
  d16 pl0:f1
  p12:sp2:f1 ph2:r

#   ifdef EXCIT
  3u
  d12
#   else
  3u
  d12 pl1:f1
  p2 ph3
  3u
#   endif /*EXCIT*/

  p16:gp1
  d16
  3u
  p16:gp2
  d16 pl0:f1
  p12:sp2:f1 ph4:r

#   ifdef EXCIT
  3u
  d12 pl1:f1
#   else
  3u
  d12 pl1:f1
  p2 ph5
  3u
#   endif /*EXCIT*/

  p16:gp2
  d16
  p1 ph1
  3u
  3u pl0:f1
  10u gron0
  (p32:sp29 ph1):f1
  20u groff
  d16 pl10:f1

                                                ;begin DIPSI2
3 p6*3.556 ph23
  p6*4.556 ph25
  p6*3.222 ph23
  p6*3.167 ph25
  p6*0.333 ph23
  p6*2.722 ph25
  p6*4.167 ph23
  p6*2.944 ph25
  p6*4.111 ph23

  p6*3.556 ph25
  p6*4.556 ph23
  p6*3.222 ph25
  p6*3.167 ph23
  p6*0.333 ph25
  p6*2.722 ph23
  p6*4.167 ph25
  p6*2.944 ph23
  p6*4.111 ph25

  p6*3.556 ph25
  p6*4.556 ph23
```

```
  p6*3.222 ph25
  p6*3.167 ph23
  p6*0.333 ph25
  p6*2.722 ph23
  p6*4.167 ph25
  p6*2.944 ph23
  p6*4.111 ph25

  p6*3.556 ph23
  p6*4.556 ph25
  p6*3.222 ph23
  p6*3.167 ph25
  p6*0.333 ph23
  p6*2.722 ph25
  p6*4.167 ph23
  p6*2.944 ph25
  p6*4.111 ph23
  lo to 3 times l1
                                                  ;end DIPSI2

  p16:gp3*-1
  d16 pl0:f1
  10u gron0*1.333
  (p32*0.75:sp29 ph1):f1
  20u groff
  d16 pl1:f1
  p1 ph6
  d0
  p1 ph1
  3u
  3u pl0:f1
  10u gron0*-1
  (p32:sp29 ph1):f1
  20u groff
  d16 pl10:f1

                                                  ;begin DIPSI2
4 p6*3.556 ph23
  p6*4.556 ph25
  p6*3.222 ph23
  p6*3.167 ph25
  p6*0.333 ph23
  p6*2.722 ph25
  p6*4.167 ph23
  p6*2.944 ph25
  p6*4.111 ph23

  p6*3.556 ph25
  p6*4.556 ph23
  p6*3.222 ph25
  p6*3.167 ph23
  p6*0.333 ph25
  p6*2.722 ph23
  p6*4.167 ph25
  p6*2.944 ph23
  p6*4.111 ph25

  p6*3.556 ph25
  p6*4.556 ph23
  p6*3.222 ph25
  p6*3.167 ph23
  p6*0.333 ph25
  p6*2.722 ph23
  p6*4.167 ph25
  p6*2.944 ph23
  p6*4.111 ph25
```

```
  p6*3.556 ph23
  p6*4.556 ph25
  p6*3.222 ph23
  p6*3.167 ph25
  p6*0.333 ph23
  p6*2.722 ph25
  p6*4.167 ph23
  p6*2.944 ph25
  p6*4.111 ph23
  lo to 4 times l2
                                                    ;end DIPSI2

  p16:gp3
  d16 pl0:f1
  10u gron0*-1.333
  (p32*0.75:sp29 ph1):f1
  20u groff
  d16 pl1:f1

  4u BLKGRAD
  p1 ph1
  go=2 ph31
  d11 mc #0 to 2 F1PH(calph(ph6, +90), caldel(d0, +in0))
exit


ph1=0
ph2=0 1 2 3
ph3=2 3 0 1
ph4=0 0 0 0 1 1 1 1 2 2 2 2 3 3 3 3
ph5=2 2 2 2 3 3 3 3 0 0 0 0 1 1 1 1
ph6=0 2
ph23=3
ph25=1
ph31=0 0 0 0 2 2 2 2


;pl0 : 0 W
;pl1 : f1 channel - power level for pulse (default)
;pl9 : f1 channel - power level for presaturation
;pl10: f1 channel - power level for TOCSY-spinlock
;sp2:wvm: userA1(cnst18 ppm, PL; PA=1.0) np=1000
;sp29: f1 channel - shaped pulse (adiabatic)
;p1 : f1 channel -  90 degree high power pulse
;p2 : f1 channel - 180 degree high power pulse
;p6 : f1 channel -  90 degree low power pulse
;p12: f1 channel - 180 degree shaped pulse
;p16: homospoil/gradient pulse                    [1 msec]
;p32: f1 channel - 180 degree shaped pulse (adiabatic)    [20 msec]
;    smoothed chirp (sweepwidth, 20% smoothing, 10000 points)
;d0 : incremented delay (2D)                   [3 usec]
;d1 : relaxation delay; 1-5 * T1
;d9 : first TOCSY mixing time (back transfer)
;d10: second TOCSY mixing time (2D)
;d16: delay for homospoil/gradient recovery
;l1: loop for first DIPSI cycle: ((p6*115.112) * l1) = mixing time
;l2: loop for second DIPSI cycle: ((p6*115.112) * l2) = mixing time
;infl: 1/SW = 2 * DW
;in0: 1/(1 * SW) = 2 * DW
;nd0: 1
;NS: 8 * n
;DS: 16
;td1: number of experiments
;FnMODE: States-TPPI, TPPI, States or QSEQ

;cnst18: = effective bandwidth for shaped pulse (ppm)
;PL: peak list to define offset(s) for shaped pulse
```

;userA1: shape function (sp2)

;phcor 2 and 4: phase difference between power levels sp2 and pl1

;choose p12 and sp2 according to desired selectivity or use WaveMaker

;for z-only gradients:

;gpz0: ca. 5%
;gpz1: 31%
;gpz2: 11%
;gpz3: 7%

;use gradient files:
;gpnam1: SMSQ10.100
;gpnam2: SMSQ10.100
;gpnam3: SMSQ10.100

```
                              ;preprocessor-flags-start
;EXCIT: for band-selective excitation start experiment with
;           option -DEXCIT (eda: ZGOPTNS)
;PRESAT: for presaturation during relaxation delay start experiment with
;           option DPRESAT (eda: ZGOPTNS)
                              ;preprocessor-flags-end
```

2D SUN-HSQC with band-selective suppression/excitation

```
;sunhsqc
;
;2D suppression of unwanted signals (SUN-HSQC)
;   using band-selective suppression or excitation
;   using DIPSI2 sequence for TOCSY transfer
;   2D H-1/X correlation via double inept transfer
;   using sensitivity improvement
;   phase sensitive using Echo/Antiecho-TPPI gradient selection
;   with decoupling during acquisition
;   with multiplicity editing during selection step
;   using shaped pulses for all 180 degree pulses on f2 - channel
;
;Use WaveMaker for definition of shaped pulses
;Define offset(s) for shaped pulses with a peak list (PL)
;The peak list must be copied from another experiment with a macro (getPL or copyPL)
;
;Band-selective suppression is default
;For band-selective excitation: Use ZGOPTNS = -DEXCIT
;
;Modified 19/08/2020
;
;           Elin Alexandersson, Corine Sandström, Lena Lundqvist and Gustav Nestor
;           Swedish University of Agricultural Sciences
;
;Avance III version
;
;$CLASS=HighRes
;$DIM=2D
;$TYPE=
;$SUBTYPE=
;$COMMENT=


#include <Avance.incl>
#include <Grad.incl>
#include <Delay.incl>


"p2=p1*2"
"d2=cnst18*p1/4"
"d4=1s/(cnst2*4)"
"d11=30m"
"d12=20u"

"spoff29=0"

"d0=3u"

"in0=inf1/2"

"DELTA=d21-cnst17*p24/2-p16-d16-p2-d0*2"
"DELTA1=p16+d16-p1*0.78+de+8u"
"DELTA2=d4-larger(p2,p14)/2"
"DELTA3=d21-cnst17*p24/2-4u"
"DELTA4=d24-cnst17*p24/2-4u"

"FACTOR1=(d9/(p6*115.112))/2"
"l1=FACTOR1*2"

"acqt0=0"
baseopt_echo


1 ze
  d2
```

```
  d11 pl12:f2
2 d1 do:f2

;dpfgse element

  d12 pl1:f1
  50u UNBLKGRAD
  (p1 ph1):f1
  3u
  p16:gp1
  d16 pl0:f1
  p12:sp2:f1 ph2:r

#   ifdef EXCIT
  3u
  d12
#   else
  3u
  d12 pl1:f1
  p2 ph3
  3u
#   endif /*EXCIT*/

  p16:gp1
  d16
  3u
  p16:gp2
  d16 pl0:f1
  p12:sp2:f1 ph4:r

#   ifdef EXCIT
  3u
  d12 pl1:f1
#   else
  3u
  d12 pl1:f1
  p2 ph5
  3u
#   endif /*EXCIT*/

  p16:gp2
  d16
  p1 ph1
  3u
  3u pl0:f1
  10u gron0
  (p32:sp29 ph1):f1
  20u groff
  d16 pl10:f1

;DIPSI2 element

3 p6*3.556 ph23
  p6*4.556 ph25
  p6*3.222 ph23
  p6*3.167 ph25
  p6*0.333 ph23
  p6*2.722 ph25
  p6*4.167 ph23
  p6*2.944 ph25
  p6*4.111 ph23

  p6*3.556 ph25
  p6*4.556 ph23
  p6*3.222 ph25
  p6*3.167 ph23
  p6*0.333 ph25
```

```
  p6*2.722 ph23
  p6*4.167 ph25
  p6*2.944 ph23
  p6*4.111 ph25

  p6*3.556 ph25
  p6*4.556 ph23
  p6*3.222 ph25
  p6*3.167 ph23
  p6*0.333 ph25
  p6*2.722 ph23
  p6*4.167 ph25
  p6*2.944 ph23
  p6*4.111 ph25

  p6*3.556 ph23
  p6*4.556 ph25
  p6*3.222 ph23
  p6*3.167 ph25
  p6*0.333 ph23
  p6*2.722 ph25
  p6*4.167 ph23
  p6*2.944 ph25
  p6*4.111 ph23
  lo to 3 times l1

  p16:gp3
  d16 pl0:f1
  10u gron0*1.333
  (p32*0.75:sp29 ph1):f1
  20u groff
  d16 pl1:f1
  p1 ph1

;hsqc element

4 DELTA2 pl3:f2
  (center (p2 ph1) (p14:sp3 ph8):f2 )
  4u
  DELTA2 pl2:f2
  (p1 ph6) (p3 ph9):f2
  d0
  (p2 ph7)
  d0
  p16:gp4*EA
  d16
  DELTA pl3:f2
  (center (p2 ph1) (p24:sp7 ph10):f2 )
  4u
  DELTA3 pl2:f2
  (center (p1 ph1) (p3 ph10):f2 )
  4u
  DELTA4 pl3:f2
  (center (p2 ph1) (p24:sp7 ph1):f2 )
  4u
  DELTA4 pl2:f2
  (center (p1 ph6) (p3 ph11):f2 )
  DELTA2 pl3:f2
  (center (p2 ph1) (p14:sp3 ph1):f2 )
  DELTA2
  (p1 ph1)
  DELTA1
  (p2 ph1)
  4u
  p16:gp5
  d16 pl12:f2
  4u BLKGRAD
```

```
  go=2 ph31 cpd2:f2
  d1 do:f2 mc #0 to 2
    F1EA(calgrad(EA) & calph(ph11, +180), caldel(d0, +in0) & calph(ph9, +180) & calph(ph8, +180) & calph(ph31, +180))
exit


ph1=0
ph2=0 1 2 3
ph3=2 3 0 1
ph4=0 0 0 0 1 1 1 1 2 2 2 2 3 3 3 3
ph5=2 2 2 2 3 3 3 3 0 0 0 0 1 1 1 1
ph6=1
ph7=0 0 2 2
ph8=0
ph9=0 0 0 0 0 0 0 0 2 2 2 2 2 2 2 2
ph10=0 0 2 2
ph11=1 1 3 3
ph23=1
ph25=3
ph31=2 0 0 2 0 2 2 0 0 2 2 0 2 0 0 2


;pl0 : f1 channel - 0W
;pl1 : f1 channel - power level for pulse (default)
;pl2 : f2 channel - power level for pulse (default)
;pl3 : f2 channel - 0W
;pl10: f1 channel - power level for TOCSY-spinlock
;pl12: f2 channel - power level for CPD/BB decoupling
;sp2:wvm: userA1(cnst18 ppm, PL; PA=1.0) np=1000
;sp3: f2 channel - shaped pulse (180 degree inversion)
;spnam3: Crp60,0.5,20.1
;sp7: f2 channel - shaped pulse (180 degree refocussing)
;spnam7: Crp60comp.4
;sp29: f1 channel - shaped pulse (adiabatic)
;p1 : f1 channel -  90 degree high power pulse
;p2 : f1 channel - 180 degree high power pulse
;p3 : f2 channel -  90 degree high power pulse
;p6 : f1 channel -  90 degree low power pulse
;p12: f1 channel - 180 degree shaped pulse
;p14: f2 channel - 180 degree shaped pulse for inversion
;     = 500 usec for Crp60,0.5,20.1
;p16: homospoil/gradient pulse
;p24: f2 channel - 180 degree shaped pulse for refocussing
;     = 2 msec for Crp60comp.4
;p32: f1 channel - 180 degree shaped pulse (adiabatic)    [20 msec]
;     smoothed chirp (sweepwidth, 20% smoothing, 10000 points)
;d0 : incremented delay (2D)                  [3 usec]
;d1 : relaxation delay; 1-5 * T1
;d4 : 1/(4J)XH
;d9 : TOCSY mixing time
;d11: delay for disk I/O                             [30 msec]
;d12: delay for power switching                             [20 usec]
;d16: delay for homospoil/gradient recovery
;d21: set d21 according to multiplicity selection
;        1/(2J(XH))  XH, XH3 positive, XH2 negative
;d24: 1/(8J)XH for all multiplicities
;     1/(4J)XH for XH
;cnst2: = J(XH)
;cnst17: = -0.5 for Crp60comp.4
;inf1: 1/SW(X) = 2 * DW(X)
;in0: 1/(2 * SW(X)) = DW(X)
;l1: loop for DIPSI cycle: ((p6*115.112) * l1) = mixing time
;nd0: 2
;NS: 16 * n
;DS: >= 16
;td1: number of experiments
;FnMODE: echo-antiecho
```

```
;cpd2: decoupling according to sequence defined by cpdprg2
;pcpd2: f2 channel - 90 degree pulse for decoupling sequence

;cnst18: effective bandwidth for shaped pulse (ppm)
;PL: peak list to define offset(s) for shaped pulse
;userA1: shape function (sp2)

;phcor 2 : phase difference between power levels sp2 and pl1
;phcor 4 : phase difference between power levels sp2 and pl1

;choose p12 and sp2 according to desired selectivity or use WaveMaker


;for z-only gradients:
;gpz0: ca. 5%
;gpz1: 31%
;gpz2: 11%
;gpz3: 7%
;gpz4: 80%
;gpz5: 20.1% for C-13, 8.1% for N-15

;use gradient files:
;gpnam1: SMSQ10.100
;gpnam2: SMSQ10.100
;gpnam3: SMSQ10.100
;gpnam4: SMSQ10.100
;gpnam5: SMSQ10.100


;cnst17: Factor to compensate for coupling evolution during a pulse
;       (usually +1). A positive factor indicates that coupling
;       evolution continues during the pulse, whereas a negative
;       factor is necessary if the coupling is (partially) refocussed.

                                        ;preprocessor-flags-start
;EXCIT: for band-selective excitation start experiment with
;             option -DEXCIT (eda: ZGOPTNS)
                                        ;preprocessor-flags-end
```
